# Molecular Mechanisms of Nicergoline from Ergot Fungus in Blocking Human 5-HT3A Receptor

**DOI:** 10.4014/jmb.2411.11020

**Published:** 2024-11-29

**Authors:** Minsu Pyeon, Myungmi Moon, Jeongyeon Yun, Jaehui Yang, Hye Duck Yeom, Gihyun Lee, Junho H Lee

**Affiliations:** 1 Department of Biotechnology and Department of Integrative Food, Bioscience and Biotechnology (BK21 FOUR), Chonnam National University, Gwangju 61186, Republic of Korea; 2 GoPath Laboratories, Buffalo Grove, IL 60089, USA; 3 Korean Medicine Research Center for Bi-Wi Control Based Gut-Brain System Regulation, College of Korean Medicine, Dongshin University, Naju-si, Jeollanam-do 58245, Republic of Korea

**Keywords:** Ergot fungus, nicergoline, open channel blocker, 5-HT3A receptor

## Abstract

This study investigates the modulatory effects of nicergoline, a major bioactive compound derived from ergot fungus, on the 5-hydroxytryptamine 3A (5-HT3A) receptor. Utilizing a two-electrode voltage-clamp technique, we evaluated the impact of nicergoline on the 5-HT-induced inward current (I_5-HT_) in 5-HT3A receptors. Our findings reveal that nicergoline inhibits I_5-HT_ in a reversible and concentration-dependent manner. Additionally, the observed voltage-dependent and use-dependent inhibition indicates that nicergoline acts as an open channel blocker of the 5-HT3A receptor. To further elucidate the interaction between nicergoline and the 5-HT3A receptor, we conducted molecular docking studies. Overactivation of the 5-HT3A receptor can enhance excitatory neurotransmission, potentially leading to heightened anxiety and stress responses. It may also interfere with hippocampal functions, adversely affecting learning and memory. Additionally, exceed activation of these receptors is a primary mechanism underlying nausea and vomiting, commonly observed during chemotherapy or in response to certain toxins. Collectively, our results suggest that nicergoline has the potential to inhibit 5-HT3A receptor activity by interacting with binding residues L260 and V264. This inhibition may enhance cognitive function by stabilizing neural circuits involved in cognitive processes and can improve cognitive symptoms in patients with dementia. Additionally, the anxiolytic effects resulting from 5-HT3A receptor inhibition could promote overall psychological well-being in affected individuals. Thus, the role of nicergoline as a 5-HT3A receptor antagonist not only highlights its therapeutic potential but also warrants further exploration into its mechanisms and broader implications for managing neurodegenerative diseases.

## Introduction

The ergot fungus, primarily found on rye and other grains, belongs to the species Claviceps purpurea. This fungus infects plants, forming small, dark brown masses known as sclerotia, which contain various bioactive compounds that can affect psychoactivity in humans [1-3]. These substances are classified as alkaloids, known for their profound effects on the central nervous system. Ergot alkaloids in ergot fungus exhibit several pharmacological actions. These alkaloids mainly bind to α-adrenergic and serotonin receptors, producing various physiological effects [4, 5]. Ergot alkaloids are known for their potent vasoconstriction properties, which can reduce blood flow. They significantly impact uterine and peripheral blood vessels and have historically been used to control postpartum bleeding [6-9].

The 5-HT3A receptor, a subtype of serotonin receptors, plays a crucial role in the central nervous system by mediating various physiological responses [10-12]. Upon activation by serotonin, these receptors are involved in regulating nausea, vomiting, and anxiety. The receptor's influence extends to the modulation of neurotransmitter release, including dopamine and acetylcholine, thereby impacting mood, cognition and visceral pain perception [13, 14]. Additionally, this receptor's involvement in the central and peripheral nervous systems underscores its significance in multiple pathways, making it a target for therapeutic interventions in conditions such as anxiety disorders, irritable bowel syndrome, and chemotherapy-induced nausea [15-18].

Ergot alkaloids affect the central nervous system, influencing sensory and cognitive functions. Some alkaloids may induce hallucinations and have historically been used in religious ceremonies for their psychoactive effects [8, 19, 20]. Nicergoline, an ergot alkaloid derivative, has various pharmacological effects include vasodilation, alpha-adrenergic receptor antagonism, and antioxidant activity, which reduce peripheral resistance and oxidative stress [21, 22]. Additionally, nicergoline offers neuroprotective benefits, potentially mitigating age-related cognitive decline [23-25]. Physiologically, it improves cognition and stabilizes hemodynamics by regulating cerebral and peripheral blood flow, making it valuable in managing symptoms of vascular dementia and neurodegeneration. Recent research on a related ergot alkaloid suggests that chanoclavine can inhibit 5-HT3A receptor activity, which may offer antiemetic effects and reduce gut stimulation [26]. Additionally, ergolines have physiological impacts, particularly in regulating the 5-HT1D receptor, which is relevant for migraine treatments [4]. These findings imply that nicergoline, sharing structural similarities with other ergot alkaloids, might demonstrate comparable effects, highlighting a possible role in serotonin-related pathways.

In this study, we suggest that nicergoline may inhibit the activity of the 5-HT3A receptor using electrophysiological recordings and molecular docking studies. We found that nicergoline modulates 5-HT3A receptor activity in a reversible, concentration-dependent, and voltage-dependent manner, resulting in varying degrees of inhibition. Additionally, we discovered that nicergoline acts as an open channel blocker of the 5-HT3A receptor, with binding residues L260 in chain D and V264 in chain E. As a subtype of serotonin receptors, the 5-HT3A receptor is involved in neurophysiological processes such as vomiting, pain, anxiety, and depression. If nicergoline inhibits this receptor, it could enhance anxiolytic and antidepressant effects, potentially alleviate pain and vomiting, and improve cognitive function. Thus, this action may offer therapeutic potential for treating various neurological symptoms in addition to its known effects on cerebral blood flow.

## Materials and Methods

### Materials

**Fig. 1:**
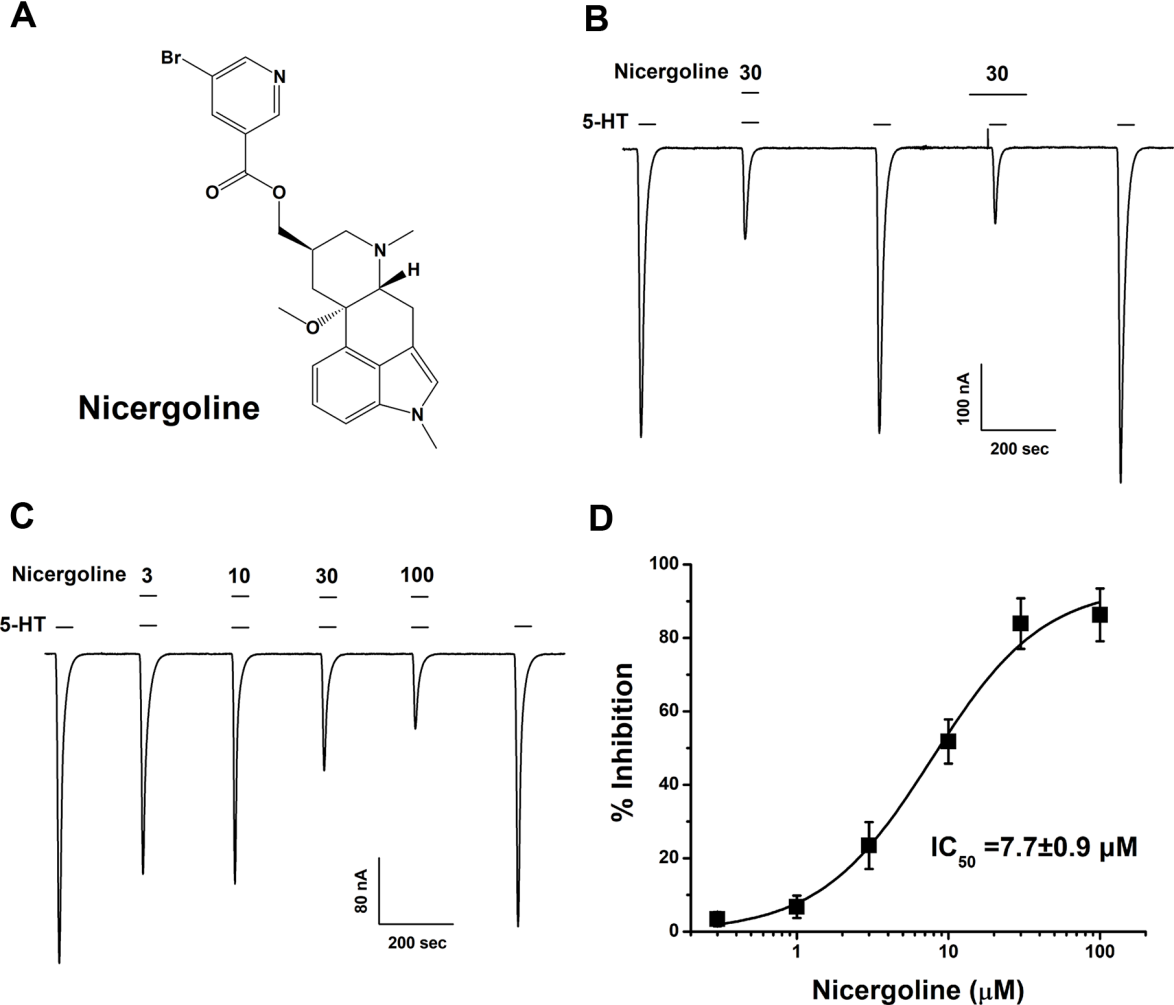
Inhibitory effects of nicergoline on 5-HT3A receptor. (**A**) The chemical structures of nicergoline. (**B**) A comparative analysis of the inhibitory effects observed when 100 μM 5-HT was co-incubated or pre-incubated with 30 μM of nicergoline on 5-HT3A receptor. Pre-incubation was performed with 30 μM nicergoline for 1 min prior to simultaneous treatment with 5-HT. To sustain the environment for each antagonist, the concentration was maintained for 1 min after 5-HT treatment. The ionic current induced by nicergoline was recorded through a two-electrode voltage clamp. Notably, the inhibitory action of nicergoline on 5-HT3A receptor-mediated currents was determined to be reversible. (**C**) The concentrationresponse relationship reflecting the co-treatment of 5-HT alongside varying concentrations of nicergoline on 5-HT3A receptor. The graph illustrates that the I_5-HT_ of 5-HT3A receptor experiences a progressive inhibition with increasing nicergoline concentrations. (**D**) The percentage of inhibition induced by nicergoline on the 5-HT inward current of 5-HT3A receptor was calculated based on the average current generated by 5-HT alone across the 5-HT3A receptor. All experiments were performed at a holding potential of −80 mV, with data presented as mean ± SEM (*n* = 9–12, derived from four distinct frogs).

We acquired the Human 5-hydroxytryptamine receptor 3A subtype plasmid DNA (Genebank number: BC004453) from OriGene Technologies, Inc. (USA). A stock solution of nicergoline (Fig. 1A) was prepared in dimethyl sulfoxide (DMSO) and then diluted in the bath medium for the experimental procedures. The final concentration of DMSO was kept below 0.05%, ensuring it did not significantly influence the reagent-induced currents. Nicergoline and all other reagents used in the experiments were sourced from Merck KGaA (Germany).

### In vitro Transcription of 5-HT3A Receptor and Site-Directed Mutagenesis

The experimental procedure follows the approach outlined in the previous study [27], but is summarized briefly as follows. The plasmid containing the cDNAs for the 5-HT3A receptor was digested with Xho I restriction enzymes to linearize the cDNAs, which were then transcribed into cRNA using the Ambion mMESSAGE mMACHINE transcription kit with T7 RNA polymerase (Thermo Fisher Scientific Inc., USA). After transcription, the cRNA was dissolved in nuclease-free water, and its size and concentration were assessed via electrophoresis and spectrophotometry (DeNovix Inc., USA). The cRNA was aliquoted to a concentration of 1 μg/μl for storage at -80°C. Specific mutations were introduced into the 5-HT3A receptor using the QuickChange site-directed mutagenesis kit (Agilent Technologies Inc., USA) and PCR. The correctness of the mutations was verified by sequencing the final mutated DNA (CosmoGenetech Co., Republic of Korea).

### Expression of 5-HT3A Receptor via RNA Microinjection and Oocyte Preparation

The study utilized female Xenopus laevis frogs that were kept according to institutional protocols (CNU IACUC-YB-2016-07, July 2016), requiring a stable temperature of around 20°C and a 12-h light-dark cycle. Frog care adhered to standards from the Korean Xenopus Resource Center for Research (KXRCR000001). Anesthesia was induced by placing the frogs on ice, after which a small abdominal incision was made to extract oocytes, which were isolated using OR2 buffer (containing 82.5 mM NaCl, 2 mM KCl, 1 mM MgCl_2_, and 5 mM HEPES, pH 7.5) with collagenase. After rinsing with ND96 buffer (which includes 96 mM NaCl, 2 mM KCl, 1 mM MgCl_2_, 1.8 mM CaCl_2_, and 5 mM HEPES, pH 7.5), stage V-VI oocytes were selected and incubated at 18°C with ND96 incubation buffer containing sodium pyruvate and gentamicin for 2 to 3 days. Finally, 40 ng of 5HT3A cRNA was injected into each oocyte using a Nanoject automatic oocyte injector.

### Molecular Protein-Ligand Docking Studies

Molecular docking studies were performed on a computer system featuring an Intel Core i9 processor at 2.20 GHz, equipped with 64 GB of RAM, and running the 64-bit version of Windows 11 (Microsoft Corp., USA). The analysis employed AutoDock Tools (version 4.2.6) from The Scripps Research Institute (USA). Structural information for the open state 5-HT3A receptor's crystal structure was sourced from the Research Collaboratory for Structural Bioinformatics (PDB ID: 8FSB), while nicergoline's three-dimensional structure was obtained from PubChem (PubChem CID: 34040, C_24_H_26_BrN_3_O_3_, MW: 484.4). Protein-ligand complexes were created to optimize binding energy, calculate inhibition constants, and analyze intermolecular interactions. LigPlot (version 4.5.3), developed by the European Bioinformatics Institute (EMBL-EBI, UK), was utilized to visualize these interactions. Additionally, the PyMOL molecular graphics system (version 2.6.0, Schrödinger Inc., USA) facilitated distance measurements and mutagenesis analysis within the 5-HT3A receptor.

### Data Recording

Two to three days post-RNA injection, the expression of the 5-HT3A protein in the oocytes was verified using a two-electrode oocyte clamp (OC-725C, Warner Instruments, LLC, USA). An agar bridge facilitated electrical connections, while ND96 buffer flowed continuously into the net chamber at 2 ml/min. Two glass microelectrodes filled with 3 M KCl (0.2 MΩ resistance) accessed the oocytes. To analyze the current-voltage relationship, voltages from −80 to +50 mV were applied, maintaining a holding potential of −80 mV. Current data were collected with a Digidata 1550 series device and analyzed using pCLAMP 10 software (Molecular Devices LLC, USA). Stock solutions of 5-HT and nicergoline were prepared at 100 mM and diluted in ND96 buffer for the experiments.

### Data Analysis

The results from the electrophysiological experiments conducted with oocytes are displayed as mean values ± standard error of the mean (SEM). To evaluate differences between the control and treatment groups for nicergoline, the Student’s t-test was employed, with a significance threshold set at *p* < 0.05. Concentration–response current data for 5-HT and nicergoline were analyzed using OriginPro (OriginLab Corp., USA) and SigmaPlot (Grafiti LLC, USA). The Hill equation was used for graph fitting in OriginPro 7.0, as detailed below:

y = V_min_ + (V_max_ − V_min_) * [x]^n^/([IC_50_]^n^ + [x]^n^)

In this formula, Vmax and Vmin represent the highest and lowest current values, respectively. IC_50_ indicates the concentration at which nicergoline achieves half-maximal inhibition. The variable x signifies the concentration of these compounds, while n corresponds to the Hill coefficient.

## Results

### Inhibitory Effects of Nicergoline on 5-HT3A Receptor

The addition of 5-HT to the bath solution produced a significant inward current in oocytes expressing the 5-HT3A receptor, demonstrating the successful functional expression of the receptor within the experimental setup. The inhibition of the 5-HT3A receptor-mediated current was evaluated using concentrations of 30 μM nicergoline. Co-treatment experiments involving nicergoline alongside 100 μM 5-HT were conducted on oocytes that expressed the cRNAs for the 5-HT3A receptor (Fig. 1B). This combination of nicergoline with 5-HT resulted in a decrease in I_5-HT_ for oocytes expressing the 5-HT3A receptor. Nicergoline exhibited similar inhibitory effects under both co-incubation and pre-incubation conditions, indicating that it does not bind to the receptor in its closed state. The pre-incubation of ligands allows for a more favorable binding environment to receptors compared to co-incubation. When this ligand acts as an antagonist, it shows greater inhibition during pre-incubation than co-incubation [28, 29]. This behavior of nicergoline suggests the possibility that it binds not to closed-state receptors but rather to receptors in the open or inactivated state.

### Concentration-Dependent Inhibition of Nicergoline on 5-HT3A Receptor

To investigate the potential variation in the inhibition rate of 5-HT3A receptor-mediated currents in response to different concentrations of nicergoline, a series of co-treatment experiments were conducted. These experiments involved administering various nicergoline concentrations alongside 100 μM of 5-HT to the 5-HT3A receptor (Fig. 1C and 1D). The results obtained from a two-electrode oocyte clamp, presented in Fig. 1D, demonstrate that increasing levels of nicergoline correspond to a greater inhibition of I_5-HT_. Half-inhibitory concentration IC_50_ for nicergoline was 7.7 ± 0.9 μM and maximum inhibition was 91.1 ± 3.4%. The Hill coefficient for nicergoline was 1.4 ± 0.2. These data showed that nicergoline exhibited inhibitory on the 5-HT3A receptor a concentration-dependent manner (*n* = 9–12 from five different frogs).

### Mechanism Studies of Inhibition on 5-HT3A Receptor by Nicergoline

**Fig. 2:**
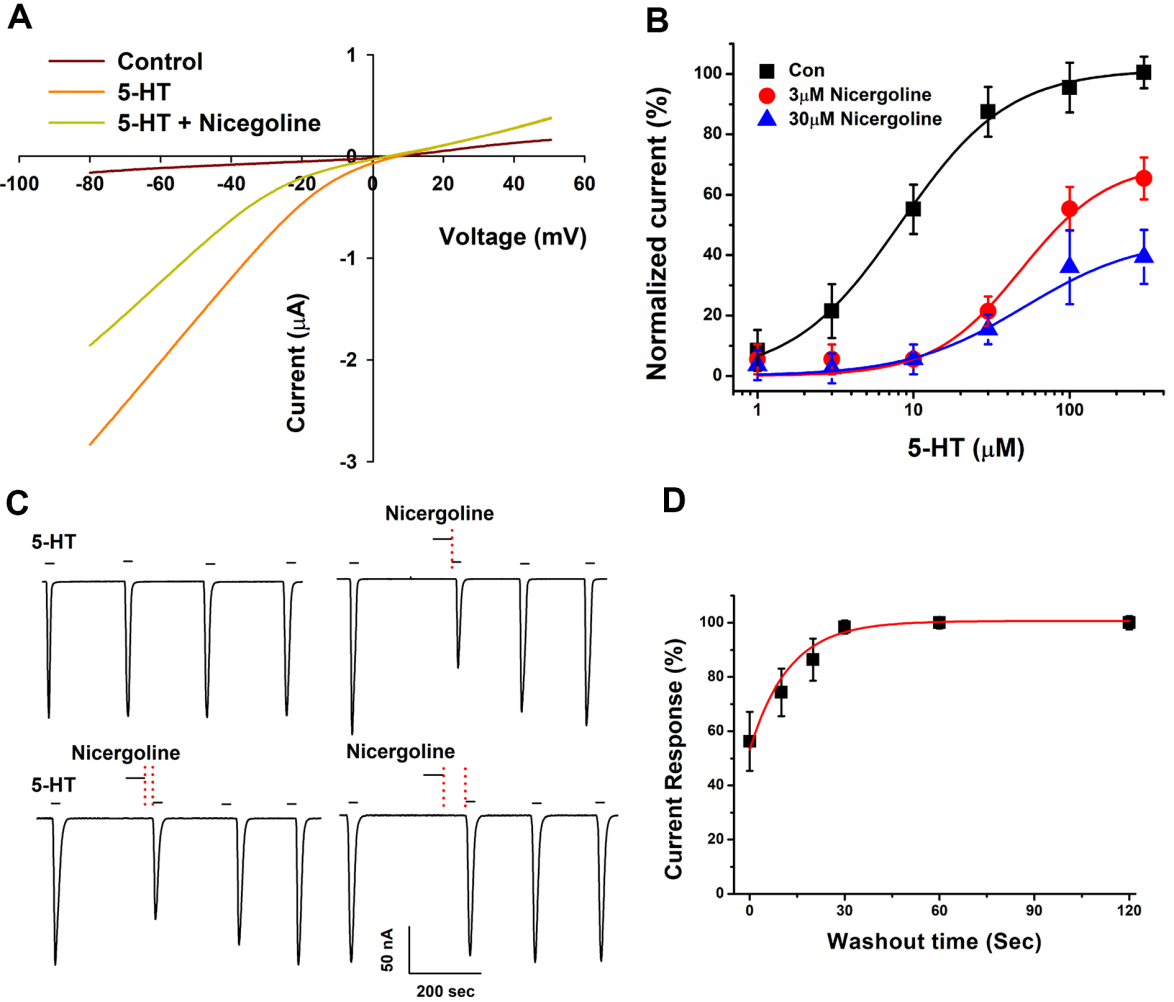
Inhibition mechanism of nicergoline on the 5-HT3A receptor. (**A**) The I_5-HT_ resulting from co-treatment with nicergoline and 100 μM 5-HT was reversible at a holding potential of −80 mV. Representative voltage-current relationship curves were obtained at holding potentials ranging from −80 to +50 mV. Voltage steps were applied to oocytes following the addition of 100 μM 5-HT, with or without 30 μM nicergoline on 5-HT3A receptor. (**B**) The I_5-HT_ was evaluated in oocytes at a holding potential of −80 mV during the co-administration of 3 or 30 μM nicergoline with varying concentrations of 5-HT on 5- HT3A receptor. The control (Con) condition involves varying concentrations of 5-HT acting on the 5-HT3A receptor without the presence of nicergoline. The curves indicated that nicergoline inhibited 5-HT3A receptor in a non-competitive fashion (*n* = 8–12 from four distinct frogs). (**C** and **D**) Recovery Times for nicergoline Inhibition. Representative traces of nicergoline application and recovery times for the 5-HT3A receptor (C, top left). In two-electrode voltage-clamp experiments, oocytes expressing the 5-HT3A receptor exhibited a stable response to repeated 100 μM 5-HT applications, with a washout period of approximately 3 min. The initial 5-HT-induced response served as the control current for recovery analysis. Nicergoline (30 μM) was administered for 2 min, followed by 5-HT application. Recovery times after nicergoline administration were 0, 10, and 30 sec (C, top right, bottom left, and bottom right). (**D**) A quantitative fitted curve representing the current amplitudes from tested traces shows that the activity of the 5-HT3A receptor was reduced to 56.2 ± 10.9% of the baseline current when 5- HT was applied immediately following nicergoline, with a subsequent stepwise recovery. Full recovery of the currents occurred after approximately 30 sec washout (*n* = 5–7, derived from four distinct frogs).

Fig. 2A demonstrates how nicergoline inhibits I_5-HT_ in the context of the voltage-current relationship for the 5-HT3A receptor. The holding potential of the membrane was set between −80 and +50 mV. Control measurements conducted in the bath solution without any treatment showed a reversal potential close to 0 mV for both the treatments with only 5-HT and those that included 30 μM nicergoline. This finding suggests that nicergoline's inhibitory effect on the inward current of the 5-HT3A receptor is dependent on the applied voltage. It is widely recognized that open-channel blockers can only bind to their sites when the channel is open, affecting either activation rates or the voltage-dependence of activation [30-32]. Our discovery that nicergoline exhibits a voltage-dependent inhibition of the 5-HT3R adds further weight to the notion that these compounds can function as open-channel blockers. This observation suggests that the inhibitory effects of nicergoline are influenced by the membrane potential, reinforcing the idea that it interacts with the receptor's open conformation. Such findings could have significant implications for understanding the mechanisms by which nicergoline and similar compounds, including certain antidepressants, modulate receptor activity, potentially informing their therapeutic applications in conditions related to serotonin signaling [28, 33, 34]. To determine whether the inhibition exerted by nicergoline is competitive with 5-HT, the concentration-response relationship of 5-HT was analyzed in the presence of 3 and 30 μM nicergoline (Fig. 2B and Table 1). All findings were normalized based on the maximum current generated by 5-HT. The resulting concentration-response curves for 5-HT in the presence of nicergoline closely mirrored those of the control but displayed notably lower normalized current values. Specifically, the presence of 3 and 30 μM nicergoline reduced the currents elicited by varying concentrations of 5-HT. The data depicted in Fig. 2B indicate that nicergoline's antagonistic effects on the activity of 5-HT at the 5-HT3A receptor are insurmountable and do not affect the potency of 5-HT, suggesting a non-competitive antagonism mechanism [35, 36]. Furthermore, the interaction dynamics between 5-HT and nicergoline with the 5-HT3A receptor imply that they bind to different sites, supporting the concept of non-competitive inhibition of 5-HT (*n* = 8–12 from four separate frogs). The reversible antagonistic effect of nicergoline on 5-HT-induced inward currents at the 5-HT3A receptor was investigated. Oocytes were exposed to 100 μM 5-HT for 30 sec, followed by episodic 5-HT applications after washing for 3 min. The agonist-induced currents stabilized (Fig. 2C, top left). After establishing a stable response, nicergoline was applied alone for 60 sec, with 5-HT applied either immediately or after 0, 10, or 30 sec (Fig. 2C, top right, bottom left, bottom right). The significant decrease in current was observed immediately following the application of nicergoline (56.2 ± 10.9% of the initial current). Recovery of current amplitude was rapid with extended wash periods. Complete recovery of 5-HT3A receptor activity occurred after approximately 51 sec washout (Fig. 2D).

### Molecular Study and Docking Model of Nicergoline and 5-HT3A Receptor

**Fig. 3:**
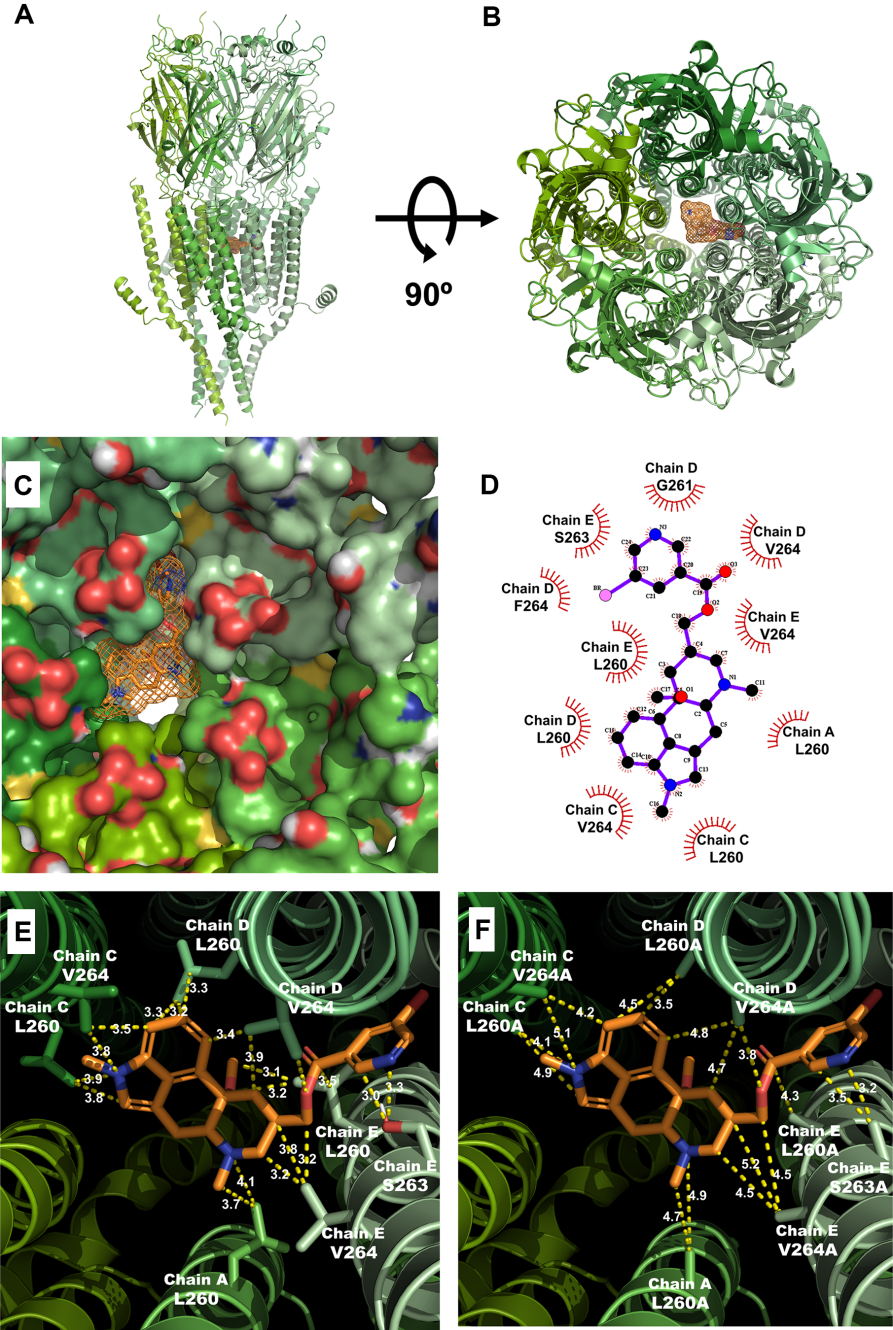
Molecular study and docking model confirmation of nicergoline interaction with the 5-HT3A receptor. (**A**) Front view of the interaction between nicergoline and the 5-HT3A receptor. (**B**) Up view of the interaction of nicergoline with the wild-type 5-HT3A receptor. The interaction is depicted with the 5-HT3A receptor represented as a tertiary protein structure, while nicergoline is modeled as a ball-and-stick representation. (**C**) Confirmation of the interaction pocket site within the 5-HT3A receptor. (**D**) The chemical interaction structure between nicergoline and the residues of 5-HT3A receptor. (**E**) Visualization of the interaction distances and involved residues between nicergoline and the wild-type 5-HT3A receptor. (**F**) Visualization of the interaction between nicergoline and the mutant 5-HT3A receptor, indicating modifications in interaction distances and involved residues.

Strategic mutations were introduced into the 5-HT3A receptor, focusing on the binding sites of both 5-HT and nicergoline, which is noted for its pronounced inhibitory effect among the tested compounds. Docking methodologies were employed to create comparative models of both the wild-type and mutant receptors. This analysis identified the most likely binding positions within the receptor's three-dimensional structure (Fig. 3A and 3B). The nicergoline binding site was found within the extracellular pore region of the transmembrane area of the 5-HT3A receptor. Fig. 3C and 3D illustrate the structure of the binding pocket for nicergoline and the positions of the amino acids involved in ligand binding. In silico modeling reinforced the idea that optimal docking results showed strong interactions between nicergoline and the wild-type receptor. AutoDock 4.0 was utilized to evaluate the activity of each residue and pinpoint the most stable binding configurations (Table 2). To confirm the interactions between nicergoline and individual residues of the 5-HT3A receptor, interaction distances were measured after each residue was mutated to alanine (Fig. 3E and 3F). In the wild-type 5-HT3A receptor, nicergoline was found to interact with the following residues: L260; distances = 3.7 and 4.1 Å in chain A, V264; distances = 3.5 and 3.8 Å, L260; distances = 3.8 and 3.9 Å in chain C, L260; distances = 3.2, 3.2 and 3.3 Å, V264; distances = 3.4, 3.5 and 3.9 Å in chain D, L260; distances = 3.1, 3.2 and 3.5 Å, S263 distances = 3.0 and 3.3 Å, V264; distances = 3.2, 3.2 and 3.8 Å in chain E. For the mutant 5-HT3A receptor, the interaction distances for nicergoline were modified as follows: L260A; distances = 4.7 and 4.9 Å in chain A, V264A; distances = 4.2 and 5.1 Å, L260A; distances = 4.1 and 4.9 Å in chain C, L260A; distances = 3.5 and 4.5 Å, V264A; distances = 3.8, 4.7 and 4.8 Å in chain D, L260A; distances = 4.3 Å, S263A; distances = 3.2 and 3.5 Å, V264A; distances = 4.5, 4.5 and 5.2 Å in chain E. These results confirm that nicergoline interacts with eight residues in the 5-HT3A receptor. The molecular interaction energies and corresponding distances between the protein and ligand are detailed in Fig. 3 and Table 2.[Table T3]

### Comparison of Nicergoline-Induced Effects on Wild-Type and Double Mutant (L260A/V264A) 5-HT3A Receptor

**Fig. 4:**
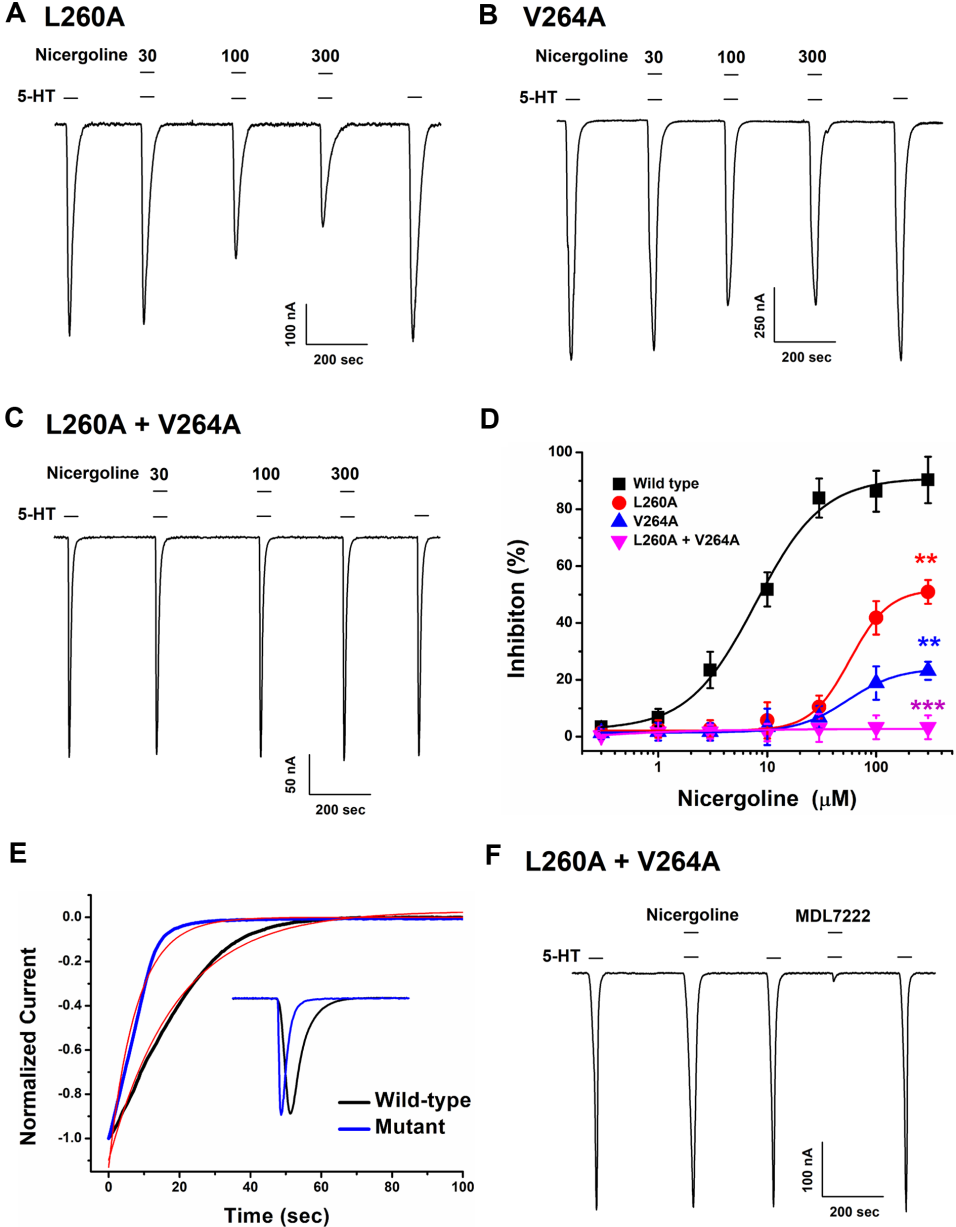
Inhibitory effects of nicergoline on double mutant 5-HT3A receptor. (**A**–**C**) The I_5-HT_ mediated by 5-HT3A receptor at a holding potential of −80 mV. Each mutant is revealing differences in the inhibitory effect of 30, 100 and 300 μM nicergoline when treated with 100 μM 5-HT. (**D**) The concentration-response graph of nicergoline on the mutant subunits, measuring 5-HT-induced inward current with 100 μM 5-HT in the presence of various nicergoline concentrations. [Wild (■), L260A (●), V264A (▲), L260A + V264A (▼)]. Data points are expressed as mean ± SEM (*n* = 7–11 from four distinct frogs). Additional parameters such as maximum inhibition, half maximal inhibitory concentration, and Hill coefficient values are detailed in Table 3. One-way ANOVA test was used for comparison comparisons between among the groups. (**p < 0.001 and ***p < 0.0001, compared with 300 μM nicergoline of the wild type. (**E**) Fast inactivation of the peak amplitude current in the double mutant (L260A/V264A) was analyzed. To investigate the rapid activation and inactivation observed in the mutant, the currents induced by serotonin in both the wild type (black line) and the mutant (blue line) were normalized and compared (E, insert). The current from peak to complete inactivation was fitted using exponential decay curve (red line) to determine the half-maximum and the maximum inactivation time. (F) To analyze the changes in antagonist activity of the double mutant (L260A/V264A), the well-known 5-HT receptor blocker MDL72222 was applied to confirm its inhibitory effect. In the double mutant (L260A/V264A), 100 μM nicergoline’s inhibitory effect was abolished, while the inhibition induced by 0.5 μM MDL72222 remained unaffected, indicating that the mutation selectively impaired nicergoline's antagonistic activity without altering the response to MDL72222.

To investigate the inhibitory properties of nicergoline on 5-HT3A receptor, point mutations were introduced into the wild-type receptor. We conducted repeated AutoDock analyses to screen for candidate binding sites, selecting approximately 30 binding clusters while excluding those located on the plasma membrane or intracellular sites. We then carried out point mutation experiments sequentially, starting with the cluster showing the most stable binding energy of nicergoline and 5-HT3A receptor. Concentration-response evaluations across various nicergoline concentrations also indicated diminished inhibition at the peak current compared to the wild-type. However, single mutations in either the L260A or V264A 5-HT3A led to lesser inhibition (51% or 24%, respectively) compared to the wild-type, and these differences were statistically significant (Fig. 4A and 4B). An amino acid within the DNA sequence of the 5-HT3A receptor was substituted with alanine, with the mutant DNAs synthesized using PCR techniques. Nicergoline exhibited approximately 91% inhibitory effect on the wild-type 5-HT3A receptor. In contrast, when assessing the double mutant comprising L260A and V264A, the inhibitory response drastically decreased relative to the wild-type (Fig. 4C). These trends were further illustrated in the graphs depicting current and percentage of inhibition (Fig. 4D). Table 2 provides a comprehensive summary of the inhibitory response values of nicergoline at the 5-HT3A receptor and mutants, indicating a clear concentration-dependent effect on I_5-HT_ based on data from multiple trials. Collectively, these findings suggest that nicergoline exerts its inhibitory effect on I_5-HT_ through interactions with the L260A and the V264A residue compare with wild type in Fig. 1C. In mutant electrophysiological study, we found the currents induced by 5-HT in the double mutant exhibited rapid activation and rapid inactivation. This indicates that when serotonin binds to the mutant, there is a swift influx of cations followed by a proper closure leading to inactivation. We compared the 5-HT induced currents between the wild type and the double mutant in Fig. 4E. The wild type (black line) and double mutant (blue line) exhibited a time to half-maximal inactivated current of 15.3 ± 1.2 sec and 7.2 ± 1.3 sec, and a time to reach maximal inactivated current of 61.9 ± 2.1 sec and 25.7 ± 3.2 sec, respectively, indicating a 2- to 3-fold faster inactivation in the double mutant than wild-type (red line). This suggests that nicergoline binds to the open channel, focusing our docking studies on the channel pore, with the mutations L260A and V264A believed to influence channel gating. To confirm whether the double mutant functions similarly to the normal channel, we examined the effects of the potent 5-HT3A antagonist, MDL 72222. While nicergoline's inhibitory effects were absent, 0.5 μM MDL 72222 strongly inhibited the 5-HT3A receptor. This indicates that the double mutant operates as a normal receptor and, like the wild type, does not affect the activity of different types of antagonists, suggesting that it represents the amino acid residues to which nicergoline binds.

## Discussion

Ergot fungus, specifically Claviceps purpurea, produces ergot alkaloids that have significant medicinal effects and applications, despite its historical associations with some side effects. The unique molecular structure of ergot alkaloids allows them to interact with receptors for neurotransmitters like serotonin and dopamine, providing therapeutic benefits in several areas. They are also applied in treating neurodegenerative conditions like Parkinson’s disease by influencing dopamine activity [7, 8]. Additionally, ergot alkaloids show potential in controlling prolactin levels, which has implications for treating certain pituitary tumors. Some ergot derivatives, like bromocriptine, are used in managing type II diabetes by impacting glucose metabolism and insulin sensitivity [37, 38]. Ergot-derived compounds are also under investigation for enhancing cognitive function, which could benefit patients with age-related cognitive decline or memory disorders [39, 40]. 

The 5-HT3 receptors play key roles in pain processing and anxiety modulation by influencing neurotransmitter release upon activation [41-43]. The 5-HT3A receptor subunit forms the main ion channel with pharmacological relevance. 5-HT3A receptor antagonists, such as ondansetron and granisetron, are extensively used as antiemetics, as they prevent serotonin-triggered vomiting by blocking these receptors in the central nervous system and peripheral nervous system [44-47]. It is known that ondansetron binds competitively to the orthosteric serotonin-binding site on the 5-HT3A receptor, preventing serotonin from activating the ion channel. Its inhibition mechanism is well-characterized as a direct blockage of receptor activation. Our research suggests that nicergoline acts as an open-channel blocker and interacts with specific residues, such as L260 and V264, within the channel pore pocket. This indicates a unique mode of inhibition that stabilizes distinct structural features of the receptor, potentially altering its conformational dynamics. A selective 5-HT3 receptor antagonist, ondansetron is highly specific to this receptor class and is mainly used for its antiemetic properties in chemotherapy, radiation, and postoperative nausea and vomiting. Its effects are focused on preventing the excitatory neurotransmission that triggers nausea and vomiting. Primarily used as an ergot derivative with multi-receptor activity, nicergoline exhibits broad pharmacological effects, including improving cerebral blood flow and neuroprotective effects. Its action on 5-HT3A receptors is part of a wider array of effects that support cognitive enhancement and neural stabilization. Additionally, 5-HT3A receptor inhibition is studied for analgesia in visceral and neuropathic pain, altering sensory processing [13, 48]. and may have anxiolytic effects by modulating GABA and dopamine release, suggesting potential applications in anxiety treatment [49-51].

These studies extensively characterize nicergoline's subtype-specific modulation of the 5-HT3A receptor, highlighting multiple mechanisms by which it influences receptor function. Electrophysiological experiments revealed that nicergoline exerts a strong, concentration-dependent inhibitory effect on I_5-HT_ currents compared to 5-HT alone. Importantly, this inhibition showed voltage dependence, suggesting that nicergoline acts as an open-channel blocker. Open-channel blockers generally interact with the receptor when the channel is in an active state, altering ion flow and stabilization patterns [28, 29, 31, 52]. In the case of nicergoline, both pre-incubation and co-incubation resulted in a similar inhibitory effect on 5-HT currents and the voltage-dependent inhibition observed indicate that nicergoline preferentially binds to the receptor in its open conformation, suggesting that it may stabilize the closed state or inhibit channel activation through interaction within the pore region. These findings align with observed behaviors in other open-channel blockers, such as certain antidepressants, which also bind to channel-specific sites distinct from the main agonist binding region. [28, 30, 53]. Nicergoline-induced inhibition of I_5-HT_ in 5-HT3A receptor-expressing oocytes was found to be voltage-dependent, within the potential range of -80 to +50 mV. Open-channel blockers are known to bind specifically when ion channels are open, influencing activation rates or voltage dependence rather than the actual opening process [30-32]. This mechanism implies that these blockers mainly obstruct ion flow without altering channel gating. Our findings show that nicergoline inhibits 5-HT3 receptors in a voltage-dependent manner, reinforcing its function as an open-channel blocker by likely interacting at the channel pore. Moreover, nicergoline's non-competitive inhibition, seen in its lack of interference with the primary 5-HT binding site, implies it targets alternative binding areas. Molecular modeling and mutational analysis identified critical residues, particularly L260 in chain D and V264 in chain E, where nicergoline binding was prominent, further supporting a non-competitive mechanism. The effects of nicergoline were reversible, indicating that its binding does not permanently alter receptor function but instead allows dynamic modulation. Despite slight variations in measured values, inhibition by nicergoline followed a consistent pattern, suggesting a reliable inhibitory mechanism that operates independently of the 5-HT agonist’s primary binding. This detailed profile suggests that nicergoline-induced modulatory role in 5-HT3A receptor function could involve selective targeting of structural regions that govern ion flow, thereby influencing receptor activity through a mechanism that avoids direct competition with 5-HT. 5-HT3A receptors are expressed on dopaminergic neurons in regions such as the mesolimbic pathway within the ventral tegmental area. Blockade of these receptors can reduce excitatory input to dopaminergic neurons, thereby modulating dopamine release in target regions like the nucleus accumbens. Additionally, 5-HT3A receptors are expressed on cholinergic interneurons, particularly in the hippocampus and cortex. Blocking these receptors can influence acetylcholine release, which is essential for cognitive processes such as attention and memory.

This study suggests that nicergoline exerts a reversible, non-competitive inhibition of I_5-HT_ at the 5-HT3A receptor, showing dependency on both concentration and voltage. Through modeling and mutational analysis, residues L260 and V264 in the 5-HT3A receptor were pinpointed as essential for nicergoline’s inhibitory effects. Electrophysiology data highlight how nicergoline interacts with specific residues to regulate the receptor channel's open state, reinforcing its potential as a unique pharmacological antagonist. These findings deepen our understanding of nicergoline's action and may guide future drug development targeting 5-HT3A receptor-related conditions.

## Figures and Tables

**Table 1:** Competition test between serotonin and nicergoline for binding to 5-HT3A receptor.

	I_max_	EC_50_	n_H_
Control	101.5 ± 4.6	8.2 ± 0.6	1.2 ± 0.1
+ 3 μM Nicergoline	70.6 ± 2.9	49.1 ± 12.4	1.3 ± 0.4
+ 30 μM Nicergoline	45.8 ± 8.6	51.3 ± 14.5	1.2 ± 0.3

Values represent means ± S.E.M. (*n* = 6–8/group). EC_50_, Hill’s coefficient; I_max_ value as determined as described in Materials and methods.

**Table 2:** The predicted channel pore docking sites and binding energy of 5-HT3A receptor and nicergoline.

	Binding energy	KI (mM)	Intermolecular energy	Internal energy	RMSD (nm)	Major binding residues
#1	-9.27	0.160	-10.76	-1.11	0.278	Asp271, Thr272, Ile267, Ile268 in chain D Asp271, Ile267 in chain E
#2	-8.62	0.480	-10.11	-1.07	0.265	L260 in chain A, L260, Val264 in chain C L260, Val264 in chain D, L260, Val264, Ser263 in chain E
#3	-8.30	0.824	-9.79	-1.57	0.262	Ser253, Glu250, Phe254 in chain C Ser253, Ile256 in chain D

*RMSD (root-mean-square deviation of atomic positions), Binding energy, Intermolecular energy, Internal energy (kcal/mol)

**Table 3 T3:** Effect of nicergoline on 5-HT evoked current of the wild-type 5-HT3A receptor and its mutants.

	Maximum inhibition	IC_50_	n_H_
Wild type	91.1 ± 3.4	7.7 ± 0.9	1.4 ± 0.2
L260A	51.9 ± 2.9	57.4 ± 5.6	1.2 ± 0.3
V264A	24.1 ± 0.8	54.7 ± 5.0	0.8 ± 0.2
L260A + V264A	2.7 ± 1.3	ND	ND

Values represent mean ± SEM. *n* = 7–11/groups. IC_50_, half-maximal inhibitory concentration of nicergoline; n_H_, Hill’s coefficient; ND, not determined
